# Neurotic Generation of Covid-19 in Eastern Europe

**DOI:** 10.3389/fpsyt.2021.654590

**Published:** 2021-07-09

**Authors:** Piotr Długosz, Liudmyla Kryvachuk

**Affiliations:** Pedagogical University of Kraków, Kraków, Poland

**Keywords:** COVID-19, neuroticism, stress, educational burnout, students, Poland and Ukraine

## Abstract

This article presents the results of a research survey, which shows the relationship between neuroticism and the coronavirus pandemic, which was performed among students in Poland and Ukraine. The survey was conducted online, on a sample of 1,978 respondents in Poland and 411 in Ukraine. The results indicated that average and high levels of neuroticism were observed among 61% of respondents in Poland and 47% in Ukraine. Regression analysis revealed that the main factors correlated with the level of neuroticism were educational burnout, gender, financial situation, interest in the pandemic, and satisfaction with life. As indicated by the respondents, neuroticism increases with educational burnout, loss of economic resources, and an increase of interest in the pandemic. Moreover, it was observed that female respondents scored higher on the scale of neuroticism compared to males. Comparative analyses between the Polish and Ukrainian students indicated that the Ukrainian youth cope with quarantine and distance education better and have better mental health. The overall responses showed that in the conditions of a pandemic, neuroticism may increase among the young generation.

## Introduction

The COVID-19 pandemic is a global macrostressor, which has a strong negative impact on the mental health of the world's population. As the coronavirus spreads, research on the psychological effects of the pandemic is gaining attention. Some researchers have applied the models and analyses that were developed during the SARS pandemic (1–3) and Swine influenza (4, 5) in their studies. An overview of the aforementioned research indicates that an increase in anxiety and depression and the emergence of posttraumatic stress disorder or the symptoms of trauma were observed among individuals due to the threat posed by those viruses and the compulsory quarantine implemented in the areas that are most affected by the epidemics (6–8).

The results clearly indicate that in addition to the threat to physical health, a deterioration of mental health is observed among populations in Germany (9), China (10), Israel (11), Russia and Belarus (12), and Turkey (13).

Deterioration of mental health among citizens due to the pandemic is expected, as the pandemic and the processes accompanying it, such as the fear of becoming infected with the coronavirus, social isolation, deprivation of needs, deterioration of economic conditions, fear of losing one's job, and financial status, highly increase the stress levels of people.

Research indicates that the younger generation is described as “coronavirus losers.” Anxiety and depression disorders can be worsened by economic stressors, the fear of poor learning results, and the stress caused by changes in daily functioning (14). A study showed that 44% of Chinese adolescents displayed symptoms of anxiety (15). Among Spanish and Italian youth, concentration difficulties were observed in 77% as reported by parents, while 55% of respondents reported boredom, 39% reported irritability, 39% reported anxiety, 33% reported nervousness, and 31% of cases reported loneliness and angst (16). In the recent studies conducted in the USA, 46% of the Generation Z respondents, 33% of the Generation X representatives, 31% of the Millenials, 28% of Boomers and 9% of older adults were convinced about the increase in stress levels due to the pandemic (17). The studies conducted among Polish youth show an increase in stress levels among them during the pandemic. It has been noted that in the age group 18–24, depression or being unhappy was observed among 26% of respondents in 2019 and 32% of respondents in 2020. The feeling of helplessness and fatigue was observed among 15% of respondents in 2019 and 44% of respondents in 2020. The feeling of weariness and lack of motivation was observed among 27% of respondents in 2019 and 47% of respondents in 2020 (18).

The fact that the pandemic is connected with the mental health of society is not unusual, but it is surprising that compared to older age groups youth suffered more severe detriments in the mental well-being, which is also indicated by numerous studies (19–21).

The above observations put forward a hypothesis that the pandemic is correlated with neuroticism among youth. The more negative the experience of the lockdown and quarantine, the higher the level of psychosomatic disorders.

It is necessary to stress that in this paper neuroticism is not understood as a personality trait (22). In the paper at hand, the interpretation of neuroticism is closely related to the ones by Karen Horney (23) and Erich Fromm (24). It is perceived as neurosis combined with social, cultural and economic factors. It is an opinion similar to modern interpretations of neuroticism as an emotional disorder (25). Caused by environmental and cultural factors as well as stressful life events (26, 27). It is worth stressing that in the Eastern European culture neuroticism is defined as a set of common symptoms of neurosis, that is, weakness, apathy, irritability, nervousness, sadness, fatigue and anxiety as well as the somatic symptoms, such as stomach ache, headache, and dizziness (28). Therefore, this notion is understood as neurotic disorders, which was removed from DSM-III (29).

## Methods

The research survey was conducted online (Computer-Assisted Web Interview) between 1st June 2020 and 10th June 2020. In Poland, the survey was conducted on a sample of 1,987 students of the Pedagogical University of Kraków, and in Ukraine on a sample of 411 students of the University of Lviv.

The selection of cities and universities was of purposive sampling nature. Metropolises on both sides of the Polish–Ukrainian border were chosen. Both cities have approximately one million inhabitants and are considered academic areas. In Kraków, there are 21 universities, in which more than 150,000 people have been studying in the 2019/2020 academic year. In turn, Lviv has 22 universities, in which 105,000 people study. In order to carry out a comparison of results between these two countries, it was decided that the research should be conducted in similar educational settings.

Therefore, a pedagogical university in Kraków, which trains future teachers, and the Faculty of Pedagogical Education of the University of Lviv were chosen for the research. This accounts for the difference in the number of respondents between the two universities.

The students who take part in the research were studying full time, were aged between 18 and 23 years, and were enrolled in undergraduate or master's degree programs. The level of religiousness was measured using an ordinal scale as follows: non-believer−1, undecided−2, believer−3, strong believer−4.

The evaluation of one's financial standing was measured using an ordinal scale by means of a question whether their financial status is worse−1, the same−2 or better−3 in comparison to other people of the same age. The population of their place of residence was measured using the following ordinal scale: village−1, city up to 20 000−2, city from 20 000 to 100 000−3, city from 100 000 to 500 000−4, city larger than 500 000−5.

The scale used to measure the levels of neuroticism was derived from the HBSC-SCL research (30), which was modified for the purpose of the Polish HBSC research (31) and adapted to the research at hand.

The neuroticism scale measured the following eight symptoms: headache; stomach ache; dizziness; trouble sleeping; nervousness; gloom, bad mood; fatigue; and irritability. The intensification of symptoms in the last 7 days was evaluated using an ordinal scale, which was developed by the authors of the research, as follows: yes, several times−4; yes, a few times−3; yes, 1–2 times−2; I haven't felt−1. The Cronbach reliability score for the Polish sample was α = 0.864 and for the Ukrainian sample was α = 0.870.

Educational burnout was defined as exhaustion due to stress and pressure which stem from the tasks and duties fulfilled by the students as regards their school and educational classes (32). The research used the LBQ Link Burnout Questionnaire (33) in the Polish version (34). This questionnaire was adapted to the needs of the present research on educational burnout during distant learning.

The scale used for measuring educational burnout had 16 items. The measurement was based on a five-point Likert scale, ranging from “I strongly agree” to “I strongly disagree.” The Cronbach reliability score for the Polish sample was α = 0.890 and for the Ukrainian sample was α = 0.863.

Satisfaction with life was measured using an ordinal scale as follows: very satisfied−1, rather satisfied−2, rather dissatisfied−3, very dissatisfied−4, and hard to say−5. The level of interest in the pandemic was measured using an ordinal scale as follows: highly interested−1, rather interested−2, rather not interested−3, and not interested at all−4. The probability of becoming infected with the coronavirus was estimated on a scale from 0 to 100%. The evaluation of distant learning was measured with an ordinal scale as follows: very good−1, good−2, average−3, bad−4, and very bad−6.

All statistical analyses were conducted using SPSS, version 25.

The respondents were selected using purposive sampling, based on their availability. A link to the survey was sent by e-mail to all the students of full-time courses in the Pedagogical University of Kraków and the students of the Faculty of Pedagogical Education of the University of Lviv.

The research is not of a representative nature. Nonetheless, it may constitute a case study and indicate how the coronavirus pandemic, the quarantine, and distant learning is correlated with the mental health of youth.

## Results

In the Polish sample Table 1, 83% of women and 17% of men participated in the survey. Female respondents were prevalent in the Ukrainian sample (97%). The statistical evaluation of the economic situation by the respondents is as follows: 17% in Poland and 16% in Ukraine evaluated the economic situation as bad; 67% in Poland and 70% in Ukraine evaluated as average; 16% in Poland and 14% in Ukraine evaluated as good. Among those surveyed, 43% of the Polish and 45% of the Ukrainian respondents were inhabitants of the countryside; 23% of the Polish and 32% of the Ukrainian respondents were inhabitants of a city having between 20,000 and 100,000 citizens; 34% of the Polish and 23% of the Ukrainian respondents were inhabitants of a big city. Regarding religiousness, 66% of participants in Poland and 88% in Ukraine declared that they had religious faith; 17% in Poland and 5% in Ukraine claimed to have no faith; 17% in Poland and 7% in Ukraine declared that they are undecided. Regarding the evaluation of distant learning, 41% of the students in Poland and 46% in Ukraine evaluated distant learning positively; 35% in Poland and 38% in Ukraine evaluated as average; 24% in Poland and 16% in Ukraine evaluated as bad. Regarding satisfaction with life, 75% of those surveyed in Poland and 81% in Ukraine declared that they were satisfied with their lives, while 15% of the Polish and 11% of the Ukrainian respondents stated that they were not satisfied. Educational burnout measured on a 16-item scale was found to be higher in Poland (M = 51.1) than in Ukraine (M = 43.8). The interest in information on the pandemic was similar between the Polish (76%) and the Ukrainian respondents (79%).

**Table 1:** Characteristics of the research sample.

		**Poland, *n* = 1.978**	**Ukraine, *n* = 411**
Age		M = 20.06 SD = 1.2	M = 19.4 SD = 1.4
Sex % (*n*)	Female	83 (1,593)	97 (395)
	Male	17 (321)	3 (8)
Financial standing % (*n*)	Bad	17 (323)	16 (66)
	Average	67 (1,279)	70 (283)
	Good	16 (307)	14 (57)
Place of residence % (*n*)	Village	43 (816)	45 (180)
	City with 20,000–100,000 citizens	23 (442)	32 (130)
	City with more than 100,000 citizens	34 (652)	23 (94)
Faith % (*n*)	Believers	66 (1,260)	88 (354)
	Non-believers	17 (322)	5 (18)
	Undecided	17 (329)	7 (32)
Evaluation of distant learning % (*n*)	Good	41 (786)	46 (192)
	Average	35 (679)	38 (154)
	Bad	24 (461)	16 (64)
Satisfaction with life % (*n*)	Satisfied	75 (1,428)	81 (326)
	Not satisfied	15 (299)	11 (46)
	Hard to say	10 (184)	8 (33)
Educational burnout	Mean (SD) Min–Max	51.1 (12.07)16–80	43.8 (10.06) 16–74
Interest in pandemic % (*n*)	Interested	76 (1,458)	79 (324)

The results showed that the responses of the Polish and Ukrainian students were similar in terms of the interest in the pandemic and the evaluation of their financial situation. However, differences were found between the two groups in their evaluation of distant learning, educational burnout, satisfaction with their lives, and religiousness. The Polish students evaluated distant learning as poor, felt exhausted, and presented worse mental well-being. On the other hand, the Ukrainian students seemed to cope with the difficulties of distant learning better. The PISA 2018 research has shown that the Ukrainian students are more satisfied with their lives than the Polish students (35). Therefore, a higher level of optimism among the Ukrainian youth is helpful in coping with the negative results of the pandemic.

For the Polish sample, the results observed on the neuroticism scale were as follows: mean value = 18.3 (SD = 5.6), median = 18, and dominant = 17. The minimum value on the scale was 8, and the maximum value was 32. Considering the obtained results, the students may be divided into three categories: those who had low levels of neuroticism—score: 8–16 (39%), medium levels—score: 17–24 (46%), and high levels—score: 25–32 (15%).

For the Ukrainian sample, the results observed on the neuroticism scale were as follows: mean value = 16.7 (SD = 5.9), median = 16, and dominant = 11. The minimum value on the scale was 8, and the maximum value was 32. Considering the obtained results, the students may be divided into three categories: those who had low levels of neuroticism—score: 8–16 (54%), medium levels—score: 17–24 (34%), and high levels—score: 26–32 (13%).

A comparison of results between the Polish and Ukrainian youth indicated that Ukrainians have a lower level of neuroticism than Poles. These differences were statistically significant, as indicated by the analysis of average results on the neuroticism scale for Polish students (M = 18.36, SD = 5.65), which are higher than those on the neuroticism scale of Ukrainian students (M = 16.74, SD = 5.96), t_(2328)_ = 5.162, *p* < 0.001, Cohen's d = 0.283.

The obtained results and the results of the research conducted during the pandemic among secondary school students in Kraków (*n* = 1,768) and Lviv (*n* = 2,291) indicate that higher levels of satisfaction were observed among Ukrainian youth (78%), compared to young Poles (66%) who were assessed on the same scale (36). This may indicate that young Ukrainians have better mental health and find it easier to cope with pandemic-related stress.

The results of variance analysis indicate that neuroticism increases in tandem with educational burnout. Psychosomatic symptoms are more common among poor students and occur more often among female students rather than males Table 2. General health is better among respondents satisfied with their lives. Students who evaluate distant learning as good have a better psychosomatic condition. It has been observed that the attitudes toward the pandemic has no impact on the average results on the neuroticism scale.

**Table 2:** Impact of independent variables on average results on the neuroticism scale – Poland.

		***n***	**M**	**SD**	
Educational burnout	Low ( ≤ 36)	227	13.2	4.63	F_(2;1, 867)_ = 318.07; *p* <0.001; η^2^ = 0.254
	Medium (37–58)	1,112	17.6	4.92	
	High (≥59)	531	22.3	4.75	
Financial standing	Worse	323	20.36	5.48	F_(2;1, 906)_ = 24.33; *p* <0.001; η^2^ = 0.025
	The same	1,279	18.09	5.50	
	Better	307	17.76	5.75	
Gender	Female	1,593	18.86	5.47	t_(1, 912)_ = 3.181; *p* = 0.001; Cohen's d is equal to = 0.48
	Male	321	16.23	5.83	
Satisfaction with life	Satisfied	1,428	17.36	5.32	F_(2;1, 908)_ = 118.52; *p* <0.001; η^2^ = 0.111
	Dissatisfied	299	21.94	5.27	
	Don't know	184	21.10	5.03	
Level of interest in the pandemic	Interested	1,458	18.48	5.66	t_(1, 911)_ =1.326; *p* = 0.185; Cohen's d is equal to 0.04
	Not interested	455	18.28	5.44	
Estimated probability of becoming infected with the coronavirus	Low	934	18.20	5.53	F_(2;1, 798)_ = 3.16; *p* = 0.043; η^2^ = 0.004
	Average	656	18.90	5.21	
	High	211	18.72	6.01	
Evaluation of distant learning	Bad	786	16.91	5.60	F_(2;1, 923)_ = 46.91; *p* <0.001; η^2^ = 0.047
	Average	679	19.17	5.30	
	Good	461	19.65	5.70	

The results of the analysis among Ukrainian students indicate that neuroticism increases together with the level of educational burnout Table 3. A worse evaluation of one's financial standing increases the levels of neuroticism. The scale of psychosomatic disorders increases together with a decrease in life satisfaction. A negative evaluation of distant learning conducted by lecturers leads to more common occurrences of symptoms of neuroticism.

**Table 3:** Impact of independent variables on average results on the neuroticism scale – Ukraine.

		***n***	**M**	**SD**	
Educational burnout	Low do ( ≤ 36)	100	13.34	5.15	F_(2;392)_ = 47.10; *p* <0.001; η^2^ = 0.194
	Medium (37–58)	271	17.16	5.44	
	High (≥59)	24	24.62	4.97	
Financial standing	Worse	65	19.90	6.05	F_(2;399)_ = 11.76; *p* <0.001; η^2^ = 0.056
	The same	281	16.23	5.74	
	Better	56	15.60	5.84	
Gender	Female	392	16.70	5.94	t_(397)_ = 0.124; *p* = 0.902; Cohen's d is equal to 0.04
	Male	7	16.42	5.59	
Satisfaction with life	Satisfied	323	15.71	5.48	F_(2;398)_ = 29.51; *p* <0.001; η^2^ = 0.129
	Dissatisfied	45	21.84	6.09	
	Don't know	33	19.84	5.70	
Level of interest in the pandemic	Interested	319	16.65	6.01	t_(401)_ = 0.627; *p* = 0.531; Cohen's d is equal to 0.07
	Not interested	84	17.11	5.76	
Estimated probability of becoming infected with the coronavirus	Low	180	16.23	5.66	F_(2;394)_ = 2.16; *p* = 0.116; η^2^ = 0.011
	Average	174	17.19	6.23	
	High	43	18.06	5.66	
Evaluation of distant learning	Good	192	15.70	5.98	F_(2;400)_ = 11.16; *p* <0.001; η^2^ = 0.053
	Average	152	16.89	5.23	
	Bad	59	19.77	6.63	

Regression analysis was performed to identify the factors that were connected with neuroticism in the research group. To this end, dependent variables were used for the assessment of sociodemographic qualities, the attitude toward distant learning, and satisfaction with life.

The results of the regression analysis indicated that a few factors may have led to neuroticism in students Table 4. Among them, educational burnout was found to be the most important. Distant learning, especially the overload of assignments, the necessity of independent learning, and lack of contacts with peers and the lecturers, led to fatigue. In other words, distant learning was a stressful and tiring process. This hypothesis was proven by the evaluation of distant learning by the Polish respondents with respect to the level of neuroticism. The worse the evaluation of distant learning, the higher was the level of neuroticism. Economic conditions also had an impact on the level of general neurosis. The students who evaluated their financial standing as worse scored higher on the neuroticism scale. This may be because the reduction of economic resources generates stress and increases neuroticism. Moreover, gender was identified as a factor related to neuroticism in the situation of the pandemic. A higher number of females displayed neurotic symptoms than males. However, this trend was observed only in the case of the Polish sample, as there were almost no males in the Ukrainian sample. The occurrence of higher levels of stress among young females during the pandemic was also indicated by the global Youth and COVID-19 research (37), and deterioration of mental health among young females was reported by recent studies (12, 38, 39).

**Table 4:** Multiple regression analyses on the neuroticism scale.

	**Poland**				**Ukraine**			
	**B**	**SE**	**β**	***P***	**B**	**SE**	**β**	***P***
Educational burnout	0.244	0.011	0.527	0.001	0.246	0.033	0.405	0.001
Financial standing	−0.482	0.201	−0.050	0.017	−1.050	0.537	−0.098	0.051
Gender	−1.934	0.305	−0.127	0.001	−1.899	2.036	−0.040	0.352
Satisfaction with life	0.905	0.109	0.174	0.001	1.343	0.284	0.247	0.001
Level of interest in the pandemic	0.173	0.143	0.029	0.031	−0.718	0.375	−0.100	0.055
Estimated probability of becoming infected with the coronavirus	0.012	0.005	0.055	0.008	0.011	0.011	0.041	0.336
Evaluation of distant learning	−0.310	0.123	−0.055	0.012	0.061	0.314	0.009	0.845
*R*^2^	0.399				0.305			
*Standard error*	4.40				4.88			
*F (p <0.001)*	161.15				26.85			

Satisfaction with life is strongly connected with neuroticism. In both Poland and Ukraine, youth who were satisfied with their lives displayed a lower level of neuroticism. Satisfaction with life seemed to serve as a defense shield, protecting youth from the adverse effects of distant learning on their mental health. The conducted analyses showed that the interest in the pandemic was also related to neuroticism. The higher the interest in the pandemic, the higher was the frequency of psychosomatic symptoms. In addition, the Polish sample indicated that an increase in fear of becoming infected was associated with higher scores on the neuroticism scale.

To sum up, it can be concluded that in both countries, the difficulties connected with distant learning and low satisfaction with life is connected with the level of neuroticism. Furthermore, all the independent variables taken into account in the analysis were correlated with neuroticism. In Ukraine, such correlations were not observed, which may result from a limited research sample.

## Discussion

Social history indicates that during a socioeconomic crisis, neuroticism is more prevalent among populations (23). Due to the Great Depression in the 1930s, the trust of people in the future collapsed and the present became bleak. The current situation appears similar. According to the conducted research, 37% of respondents in Poland and 45% in Ukraine have lost a sense of control over their own lives due to the pandemic. Almost half of the youth in both countries have suffered a severe shock and their lives have become unpredictable. The loss of existential security is accompanied by educational burnout. On one hand, the future is uncertain and threatening, as the second wave of infection is expected in the autumn. The resulting economic crisis and unemployment may prevent youth from successfully entering adulthood at a natural pace, which is demonstrated by the results of this research survey. On the other hand, the present situation is bad and stressful. The students are not afraid of becoming infected with the coronavirus, as the probability has been estimated at ~38% in both countries, but they are stressed with the quarantine, deterioration of economic conditions, monotony, loneliness, and distant learning. The second round of the survey conducted among the Polish students indicated that after 3 months of quarantine, the deterioration of mental health and increase in negative emotions such as anxiety, loneliness, exhaustion, anger, and psychosomatic symptoms materialized (40). Similar results indicating an increase in the levels of fear, anxiety and stress have been obtained from young respondents in Spain (41), Cyprus (42), China (43), the USA (44) and France (45). All of the above studies indicate that distant learning, quarantine and the lack of peer contacts lead to a deterioration of the respondents' mental health.

The deprivation of the need for security, belonging, acceptance, and self-actualization (46) in the conditions of quarantine induce pandemic-related stress. It is worth indicating that the studies into cohorts of youth in the USA indicate that in the recent years, the levels of fear and neuroticism have increased (26). The author maintains that civilisation changes are responsible for the deterioration of mental health. Anxiety increases together with the increase of environmental threat, deterioration of one's financial situation and the weakening of social bonds. All of these factors occur during the pandemic crisis. We face a threat which cannot be controlled, and distant learning weakened or limited social contacts. What is more, as indicated by the results of research conducted in the recent years, a significant negative correlation between the use of Digital media and mental health has been observed (47). It may be assumed that together with the pandemic and the imposition of distant learning in Poland and Ukraine, educational burnout started occurring at a mass scale. Educational burnout becomes a neuroticism risk factor among youth.

As stated by Karl Mannheim (48), it can be accepted that at the time of crisis and radical social changes, a generation is forming. A major factor that is correlated with the formation of a generation is trauma experienced during a historic event. Perhaps, the COVID-19 pandemic is one such generational event. The pandemic has resulted in shock and present traumas, while also giving rise to secondary trauma. The generational history (49) has made a circle, and we are once again in a deep crisis which induces neurotic behaviors among the younger generation.

### Limitations

The present study has certain limitations. Since data were collected from participants on a voluntary basis through an online application, generalizations should be cautiously made. The measurement of the variables: satisfaction with life, interest in the pandemic, probability of becoming infected and distance education was performed with the use of a single-item response scale and a response scale consisting of a few options. The research sample includes an over-representation of females in comparison to few male respondents, which was particularly observed in Ukraine. This may affect the results of research. While interpreting the results, one should bear in mind those methodological limitations.

Only after years have passed, it would be possible to determine the influence of historic events on the attitudes of youth. Therefore, the results of the present study should be perceived as a direction for future research on how the pandemic is correlated with the mental condition of youth in Eastern Europe.

## Data Availability Statement

The raw data supporting the conclusions of this article will be made available by the authors, without undue reservation.

## Ethics Statement

The studies involving human participants were reviewed and approved by Rector's Committee for Ethics of Scientific Research. The ethics committee waived the requirement of written informed consent for participation.

## Author Contributions

PD and LK contributed to the design and implementation of the research, to the analysis of the results and to the writing of the manuscript. Both authors contributed to the article and approved the submitted version.

## Conflict of Interest

The authors declare that the research was conducted in the absence of any commercial or financial relationships that could be construed as a potential conflict of interest.
